# Choledochal cyst as an incidental finding during acute cholecystitis: A case report

**DOI:** 10.1002/ccr3.8515

**Published:** 2024-02-09

**Authors:** Ryan Isaac Sia Zu Wern, Pravallika Venna, Akash Sarkar, Marwa Abdul‐Haque, Saachi Bhattessa

**Affiliations:** ^1^ GKT School of Medical Education, King's College London Guy's Campus London UK; ^2^ SVS Medical College Mahabubnagar India; ^3^ Faculty of Medicine University of Debrecen Budapest Hungary; ^4^ Faculty of Life Sciences and Education University Of South Wales Cardiff UK; ^5^ Vinnytsia National Medical University Vinnytsia Oblast Ukraine

**Keywords:** adults, biliary, CDCs, choledochal cysts

## Abstract

**Key Clinical Message:**

This case demonstrates an atypical presentation of choledochal cysts (CDCs) and elaborates on the diagnostic challenges encountered when presented with CDCs in adulthood, as it principally presents in children.

**Abstract:**

A *choledochal cyst* is a rare congenital anomaly characterized by cystic dilations in the extrahepatic and intrahepatic biliary trees. These cysts are classified according to their location and characteristics. This case study aims to demonstrate how nonspecific clinical features can pose a diagnostic dilemma when presented in adults. Additionally, the case report provides an overview of diagnostic methods and treatment options. In this case, we discuss a 50‐year‐old female who presented with a 2‐ to 3‐day history of severe colicky pain in the right upper quadrant of her abdomen without any other symptoms or abnormal laboratory tests. In addition to ultrasonography evidence of CBD dilation and cholelithiasis, MRCP results confirmed the diagnosis. She underwent surgical intervention involving cyst excision, a Roux‐en‐Y hepatojejunostomy, and a cholecystectomy. The postoperative period was without significant complications. The case presented here illustrates the potential outcomes for individuals who present with choledochal cysts during adulthood. Often, these cases present with vague symptoms or as the underlying cause of a more severe condition. This case contributes to the existing knowledge of choledochal cysts by providing insight into the clinical presentation, diagnostic methods, and treatment options.

## 1 INTRODUCTION

Choledochal cysts (CDCs) are a rare premalignant condition characterized by cystic dilation of the biliary tree, either intra or extrahepatic.^1^ Although their exact etiology is still unknown, these are congenital abnormalities that are thought to arise from pancreaticobiliary maljunction leading to the formation of a long common channel.^1^ This allows the reflux of pancreatic secretions due to higher secretory pressures in the pancreas compared to the biliary tract, causing the mixing of their contents and the activation of pancreatic enzymes.^2^ The consequent inflammation and high intraductal pressures lead to cystic changes and the formation of CDCs. Subsequent changes may include dysplasia due to chronic inflammation if left untreated, and an elevated risk of cholangiocarcinoma and gallbladder carcinoma, with a lifelong risk of up to 4% even following cyst excision.^3^ Cholelithiasis may develop due to biliary stasis, obstructing the extrahepatic biliary tract, causing ascending cholangitis and secondary biliary cirrhosis.

Diagnosis is based on clinical symptoms and imaging studies, with ultrasonography being the initial modality of choice, but CT can often provide better visualization of the terminal CBD, such as in the case of bowel gas. However, magnetic resonance cholangiopancreatography (MRCP) and endoscopic retrograde cholangiopancreatography (ERCP) are used for confirmatory diagnosis, with MRCP being the preferred modality due to its higher sensitivity and safety as a noninvasive procedure.^4^

CDCs are usually diagnosed during childhood, with only about 20% diagnosed past the age of 20.^5^ Pediatric and adult patients present differently, with obstructive jaundice, palpable abdominal mass, and sudden severe obstruction of the terminal CBD, more commonly associated with pediatric patients.^6^ Both patients typically present with abdominal pain, but CDCs can also have atypical clinical presentations in adults and thus pose diagnostic challenges, as in the following case report.

## 2 CASE HISTORY

A 50‐year‐old female presented with a history of pain in the right hypochondrium associated with bilious vomiting for the past 3 days.

## 3 METHODS (DIFFERENTIAL DIAGNOSIS, INVESTIGATIONS, TREATMENT)

**FIGURE 1:**
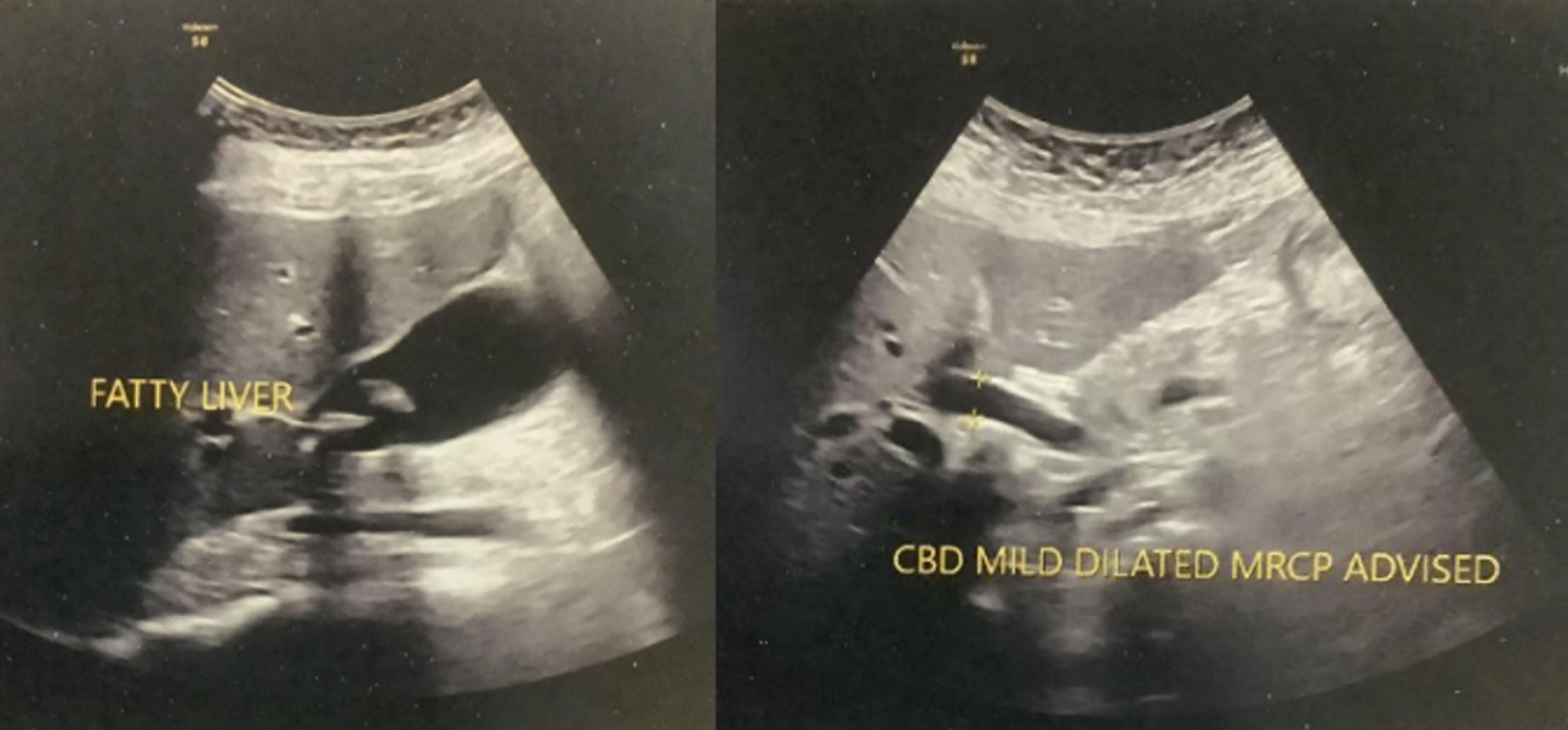
Ultrasonography scans of the patient.

Laboratory findings included an increase in neutrophils to 91% (Normal: 50–70) and a decrease in the lymphocytes to 5% (Normal: 20–46) with a normal white blood cell count. Liver function tests were within a normal range. Ultrasonography (Figure 1) revealed multiple cholelithiasis with a mildly over‐distended gall bladder and mildly thickened walls. A calculus of 12 mm was impacted at the neck of the gall bladder. The common bile duct was mildly dilated (11‐12 mm) with prominent hepatic ducts. No calculus was present in the visualized part of CBD.

A diagnosis of choledochal cyst was suspected, and MRCP was advised. MRCP showed the presence of multiple cholelithiasis with a dilated common bile duct measuring about 11 mm. The patient underwent a choledochal cyst excision with Roux‐en‐Y hepaticojejunostomy with cholecystectomy. The gall bladder measured 8 × 5 × 4 cm with a wall thickness of 0.4 cm. The external surface of the gall bladder was congested, and the cut surface showed ulcerated mucosa. Multiple calculi were visible. A single gray‐white tissue measuring 2.5 × 3 cm was obtained to be biopsied. The microscopic picture showed inflammation in the wall of the choledochal cyst and was also suggestive of chronic cholecystitis. No inflammation of the common bile duct was noticed.

## 4 CONCLUSION AND RESULTS (OUTCOME AND FOLLOW UP)

The postoperative period was uneventful, and the patient was discharged with stable vitals along with the following medications: ofloxacin, pantoprazole, laxatives and Dynapar (diclofenac and paracetamol). On follow‐up, after 7 days, repeat ultrasonography was performed, which showed mild postsurgical pneumobilia and post‐cholecystectomy status without any definitive collection.

## 5 DISCUSSION

First described by Vater and Ezler, choledochal cysts (CDCs) are abnormal congenital cystic dilatations of the biliary tract. Although rare among Western populations (~1 in 100,000–150,000), it disproportionately affects Asian populations (~1 in 1000).^7^ Most cases of CDCs present in infancy and childhood, with only about 20% of cases presenting in adulthood, with a female predominance of 4:1 to 3:1.^8^, ^9^

**FIGURE 2:**
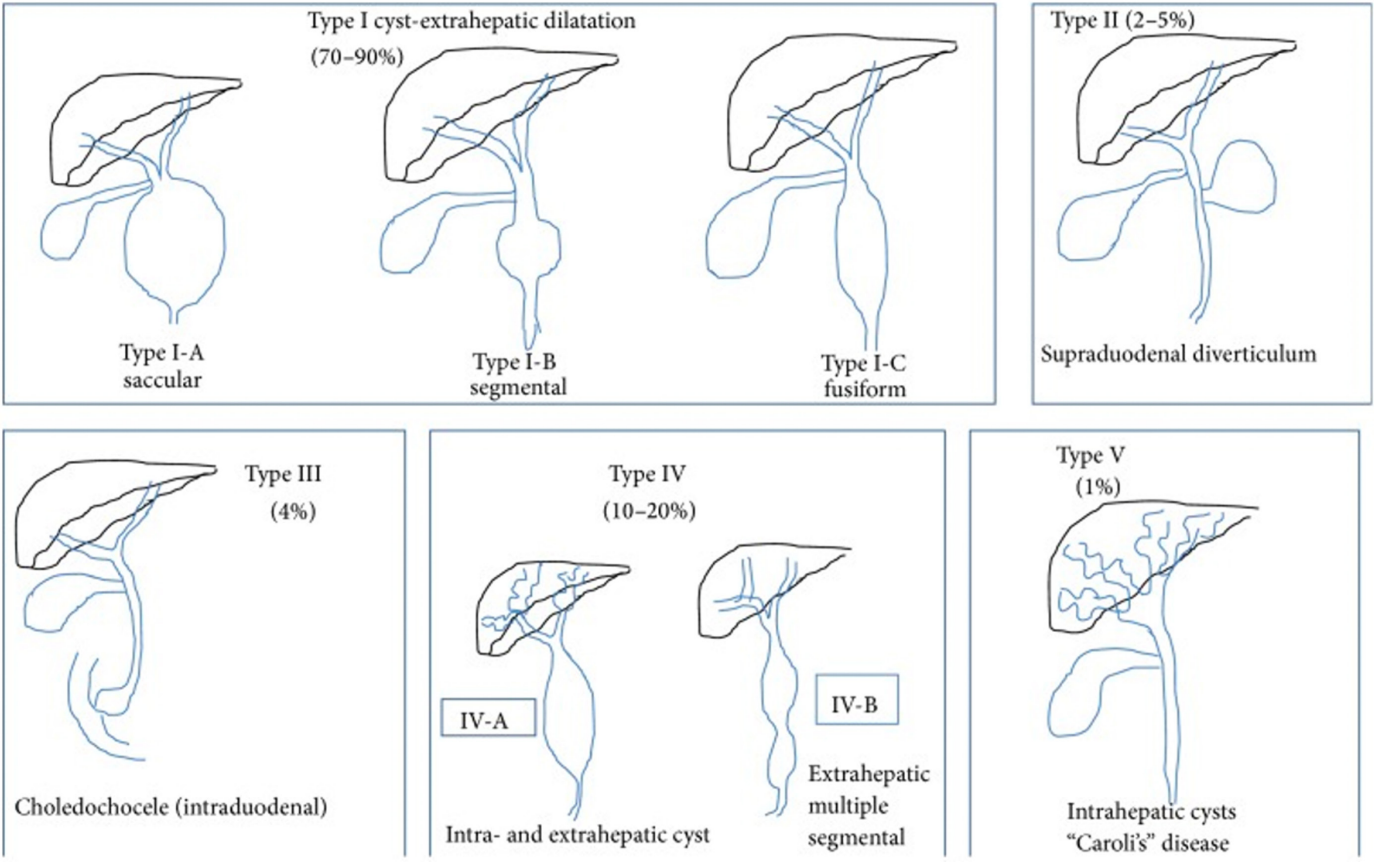
Todani's classification of choledochal cysts.

The most widely accepted classification of choledochal cysts among clinicians was established by Todani et al. whose breakdown into five subtypes expanded upon Alonso‐Lej's initial work, with the inclusion of both multiple (Type IV) and intrahepatic cysts (Type V).^7^ Type 1 choledochal cysts are extrahepatic cysts and make up the most common type of CDCs (80%).^10^ This is broken down into three further subdivisions, with the overarching principle being a dilatation of the common bile duct (CBD) with normal intrahepatic ducts. The subdivisions include: (1a) diffuse dilatation of the CBD, (1b) isolated dilatation of the distal CBD, and (1c) fusiform CBD dilatation, which the patient had presented with.^10^ This is illustrated by Figure 2 below.

Although its etiology remains unknown, the most widely accepted theory was postulated by Babbitt et al. whose work suggested an anomaly of the pancreaticobiliary ductal system during embryonic development predisposed to the formation of CDCs.^12^ In particular, the junction of the CBD and pancreatic duct occurs outside of the ampulla of Vater at a right angle, favoring the retrograde reflux of bile and pancreatic contents up the ductal system.^12^, ^13^ Consequently, a long common channel of the Sphincter of Oddi forms (>15 mm).^8^, ^13^ Over time, the reflux of caustic pancreatic enzymes causes chronic inflammation, damaging the biliary epithelium and, ultimately, leading to the formation of CDCs.^13^ Thus, when diagnosed in adulthood, CDCs at biopsy often highlight mucosal ulceration and hyperplasia, correlating with the histopathology findings of the patient's resected gallbladder and CDC.^1^

Studies have shown that this anomalous pancreaticobiliary duct union accounts for 30%–70% of CDCs at diagnosis, increasing up to 96% in the pediatric population.^8^ The suggested cause of a fusiform CBD dilatation (Type 1c), which was measured at 11–12 mm on ultrasonography, is the chronic reflux of pancreatic enzymes, linking with Babbitt et al. and their proposed pathogenesis of CDCs.^12^, ^13^ Furthermore, the patient's presentation of severe colicky pain, together with a history of intense dieting with minimal water consumption over the last few years, explains the findings gathered; the 12 mm calculi at the neck of the gallbladder, alongside asymptomatic bilateral nephrolithiasis on ultrasonography.

When investigating a patient with unexplained right upper quadrant pain, ultrasonography is the preferred first‐line imaging modality. Not only is it a useful imaging modality in emergency scenarios due to its accessibility, but it has a sensitivity of 71% to 97% for identifying and localizing CCs.^1^ Although ultrasonography remains a good imaging modality, it is known that if a CBD dilatation is found, it fails to identify the underlying cause in over 30% of patients, like this patient.^1^, ^8^ Indeed, a CBD measuring >10 mm on ultrasonography should indicate that a cystic dilatation or an obstruction has occurred.^8^ This should prompt the use of other imaging modalities such as MRCP and ERCP, which both possess higher sensitivities (70%–100%) and specificities (90%–100%) for diagnosis of CDCs, with MRCP preferred for its reduced rate of complications.^1^, ^8^, ^9^

The established gold standard treatment for Type 1 CDC is excision of the CDC with cholecystectomy, after which bile flow is restored by hepaticoduodenostomy (HD) or Roux‐en‐Y hepaticojejunostomy (RYHJ).^8^ The patient underwent a CDC excision with RHYJ and cholecystectomy. As reported in literature, an RYHJ has more favorable outcomes to an HD across a spectrum of measures.^8^, ^9^ These are not limited to decreased rates of gastric and biliary cancer and significantly decreased rates of postoperative reflux.^8^, ^9^

Importantly, this case report highlights an atypical presentation of a CDC and underlines the diagnostic difficulty when CCs are present in adults. An increase in reported CDC cases in adults in recent years, with more than 70% of cases attributed to adults, necessitates greater awareness, especially since it may be frequently overlooked.^1^, ^14^ Without typical features of CDCs, which are more commonly seen in children, such as the classic triad of pain, jaundice, and an abdominal mass (85% of children present with 2/3 clinical features compared to 25% in adults), diagnosis heavily relies on imaging modalities, alongside clinical acumen.^14^, ^15^ The patient, who had unremarkable systemic findings at presentation, alongside normal LFTs and serum amylase and lipase markers, emphasizes this. However, this is not a rarity given that CDCs are frequently only first suspected in adults when presenting with vague upper abdominal pain.^14^

The importance of prompt diagnosis of CDCs to achieve optimal patient outcomes in adults is underlined by the complications arising from untreated CDCs, which are often seen as pre‐malignant.^16^ The chronic inflammatory process is thought to induce dysplastic changes, ultimately leading to CC‐related carcinomas—a well‐known long‐term complication of untreated CDCs in adults.^8^ This risk reaches up to 50% when CDCs are found in 51‐ to 70‐year‐old patients.^17^ Other complications of CDCs, which may be present on initial presentation, include ascending cholangitis, biliary cirrhosis, pancreatitis and portal hypertension.^8^, ^9^, ^16^, ^17^

Even with prompt surgical intervention, the risk of postoperative complications remains. Despite the patient's follow‐up showing no immediate complications, except for mild pneumobilia, long‐term follow‐up will be required. Whilst the risk of anastomotic strictures is reduced with a high anastomosis (RHYJ), malignancy can develop years after CDC excision, with late complications (>30 days) seen in over 40% of adults.^1^, ^9^ It goes without saying that the long‐term prognosis of resected CDCs in adults remains excellent, with more than 85% of patients having no long‐term complications, but regular monitoring is essential to detect potential biliary malignancy.^8^

## 6 CONCLUSION

This case demonstrates an atypical presentation of CDCs and elaborates on the diagnostic challenges encountered when presented with CDCs in adulthood, as it principally presents in children. Due to the recent increase in the prevalence of CDC in the adult population, it is essential to keep CDCs as a differential diagnosis to prevent complications from developing.

## AUTHOR CONTRIBUTIONS

**Ryan Isaac Sia Zu Wern:** Conceptualization; data curation; resources; software; writing – original draft; writing – review and editing. **Pravallika Venna:** Investigation; methodology; visualization; writing – original draft. **Akash Sarkar:** Formal analysis; supervision; validation; writing – original draft. **Marwa Abdul‐Haque:** Methodology; project administration; writing – original draft; writing – review and editing. **Saachi Bhattessa:** Project administration; validation.

## FUNDING INFORMATION

The author(s) received no financial support for the research, authorship, and/or publication of this article.

## CONFLICT OF INTEREST STATEMENT

The author(s) declared no potential conflicts of interest with respect to the research, authorship, and/or publication of this article.

## ETHICS STATEMENT

Ethical approval was not needed with respect to a case report by our institution.

## CONSENT

Written informed consent was obtained from the patient for her anonymized information to be published in this article. The patient regained fair insight and judgment over the course of her treatment regime to provide written informed consent by herself.

## Data Availability

Data sharing not applicable—no new data generated, or the article describes entirely theoretical research.

## References

[1] Koga H , Yamataka A . Choledochal cyst. Pediatric Surgery. Vol 29. Springer; 2023:1101‐1115. doi:10.1007/978-3-030-81488-5_80 37261604

[2] Song HK , Kim MH , Myung SJ , et al. Choledochal cyst associated the with anomalous union of pancreaticobiliary duct (AUPBD) has a more grave clinical course than choledochal cyst alone. Korean J Intern Med. 1999;14(2):1‐8. doi:10.3904/KJIM.1999.14.2.1 PMC4531926 10461418

[3] Madadi‐Sanjani O , Wirth TC , Kuebler JF , Petersen C , Ure BM . Choledochal cyst and malignancy: a plea for lifelong follow‐up. Eur J Pediatr Surg. 2019;29(2):143‐149. doi:10.1055/S-0037-1615275 29258149

[4] Khandelwal C , Anand U , Kumar B , Priyadarshi RN . Diagnosis and Management of Choledochal Cysts. Indian J Surg. 2012;74(1):29‐34. doi:10.1007/S12262-011-0388-1 23372304 PMC3259169

[5] Atkinson HDE , Fischer CP , De Jong CHC , Madhavan KK , Parks RW , Garden OJ . Choledochal cysts in adults and their complications. HPB (Oxford). 2003;5(2):105‐110. doi:10.1080/13651820310001144 18332966 PMC2020569

[6] Huang CS , Huang CC , Chen DF . Choledochal cysts: differences between pediatric and adult patients. J Gastrointest Surg. 2010;14(7):1105‐1110. doi:10.1007/S11605-010-1209-8 20422306

[7] Singham J , Yoshida EM , Scudamore CH . Choledochal cysts: part 1 of 3: classification and pathogenesis. Can J Surg. 2009;52(5):434. 19865581 PMC2769090

[8] Soares KC , Arnaoutakis DJ , Kamel I , et al. Choledochal cysts: presentation, clinical differentiation, and management. J Am Coll Surg. 2014;219(6):1167‐1180. doi:10.1016/J.JAMCOLLSURG.2014.04.023 25442379 PMC4332770

[9] Bhavsar MS , Vora HB , Giriyappa VH . Choledochal cysts: a review of literature. Saudi J Gastroenterol. 2012;18(4):230‐236. doi:10.4103/1319-3767.98425 22824764 PMC3409882

[10] Todani T , Watanabe Y , Narusue M , Tabuchi K , Okajima K . Congenital bile duct cysts: classification, operative procedures, and review of thirty‐seven cases including cancer arising from choledochal cyst. Am J Surg. 1977;134(2):263‐269. doi:10.1016/0002-9610(77)90359-2 889044

[11] Machado NO , Chopra PJ , Al‐Zadjali A , Younas S . Choledochal cyst in adults: etiopathogenesis, presentation, management, and Outcome‐case series and review. Gastroenterol Res Pract. 2015;2015:602591. doi:10.1155/2015/602591 26257778 PMC4518150

[12] Babbitt DP , Starshak RJ , Clemett AR . Choledochal cyst: a concept of etiology. Am J Roentgenol. 1973;119(1):57‐62. doi:10.2214/ajr.119.1.57 4744730

[13] Craig AG , Chen LD , Saccone GTP , Chen J , Ta Padbury R , Toouli J . Sphincter of Oddi dysfunction associated with choledochal cyst. J Gastroenterol Hepatol. 2001;16(2):230‐234. doi:10.1046/J.1440-1746.2001.02365.X 11207909

[14] Söreide K , Körner H , Havnen J , Söreide JA . Bile duct cysts in adults. Br J Surg. 2004;91(12):1538‐1548. doi:10.1002/BJS.4815 15549778

[15] Lipsett PA , Pitt HA , Colombani PM , Boitnott JK , Cameron JL . Choledochal cyst disease a changing pattern of presentation. Ann Surg. 1994;220(5):644‐652. 7979612 10.1097/00000658-199411000-00007 PMC1234452

[16] Nagorney DM , McIlrath DC , Adson MA . Choledochal cysts in adults: clinical management. Surgery. 1984;96(4):656‐663. Accessed August 10, 2023. http://www.surgjournal.com/article/0039606084903088/fulltext 6091285

[17] Nicholl M , Pitt HA , Wolf P , et al. Choledochal cysts in western adults: complexities compared to children. J Gastrointest Surg. 2004;8(3):245‐252. doi:10.1016/J.GASSUR.2003.12.013/METRICS 15019916

